# The Research and Applications of Quantum Dots as Nano-Carriers for Targeted Drug Delivery and Cancer Therapy

**DOI:** 10.1186/s11671-016-1394-9

**Published:** 2016-04-18

**Authors:** Mei-Xia Zhao, Bing-Jie Zhu

**Affiliations:** Key Laboratory of Natural Medicine and Immune Engineering, Henan University, Kaifeng, 475004 China

**Keywords:** Quantum dots, Nano-medicine, Targeted drug delivery, Biomaterial

## Abstract

Quantum dots (QDs), nano-carriers for drugs, can help realize the targeting of drugs, and improve the bioavailability of drugs in biological fields. And, a QD nano-carrier system for drugs has the potential to realize early detection, monitoring, and localized treatments of specific disease sites. In addition, QD nano-carrier systems for drugs can improve stability of drugs, lengthen circulation time in vivo, enhance targeted absorption, and improve the distribution and metabolism process of drugs in organization. So, the development of QD nano-carriers for drugs has become a hotspot in the fields of nano-drug research in recent years. In this paper, we review the advantages and applications of the QD nano-carriers for drugs in biological fields.

## Review

Nowadays, many anti-tumor drugs in clinical tests have various side effects, such as non-selective, toxicity, poor targeting, etc. [1]. So, the heavy side effects of normal tissues produced by a conventional therapeutic dose of anti-tumor drugs would reduce drug efficacy [2]. Severe side effects arise from the use of classical cancer therapeutics due to non-specificity of the cancer drugs, resulting in high toxicity in noncancerous but rapidly dividing cells [3]. Due to the lack of any bioluminescence, tumors can be difficult to be located and removed, particularly on smaller size scales. Therefore, it is highly desirable to create a means by which cancer cells could be targeted with high specificity and be simultaneously imaged in vivo [4]. Nano-carriers for drugs mainly have nano-vehicles (including liposomes, etc.), polymer nanoparticles/nano-capsules, microemulsion/nano-emulsion, micelles, dendrimers, and inorganic nano-carriers (such as silica nano-spheres, nano-tubes, and quantum dots [QDs]) [5, 6]. QDs possess unique optical properties that make them potential candidates as luminescent nano-probes and carriers for biological applications. Drugs can be loaded into QD nano-carriers for drugs by the means of dissolving, dispersing, adsorption and coupling, etc. [5, 7]. Then, the physical and chemical properties (such as saturation solubility, dissolution rate, crystal form, particle surface hydrophobicity, and hydrophilicity), physical response, and biological characteristics of drugs are changed due to the role of the carriers; thus, the absorption, distribution, metabolism, and excretion of drugs are affected [8]. Ultimately, QD nano-carriers for drugs can enhance the efficacy and reduce side effects of drug reactions to improve the therapeutic index of the drugs [9]. Moreover, nano-carriers for drugs can effectively promote the absorption of small molecule drugs. At the same time, the research about macromolecular drug delivery has also demonstrated good prospects [10].

As a new kind of inorganic nano-fluorescent probe, QDs have showed outstanding advantages in the long-time, multi-color fluorescence imaging and detection [11, 12]. The development of QD labeling promotes the research in the nano-drugs in cellular, even at live animal level. The development of fluorescence imaging technology of QDs and the therapy-based multifunctional nano-drugs is expected to apply to diagnosis and treatment of cancers [13, 14]. At the same time, surface modifications with targeting ligands are also commonly used to increase drug-delivery efficiency [15]. After almost 10 years of development, the technology of QDs surface modifications has realized relative improvements. The surface ligands can be thioglycolic acid, cysteamine or polyethylene glycol (PEG), and water-soluble polymers with carboxyl, which can bind the drug molecules with QDs through electrostatic binding or covalent bond and form nano-drug complexes carriers, and then realize fluorescent trace of drug molecules in cells or animals [16].

### Advantages of QDs as Nano-Carriers in Targeted Drug Delivery

Compared with conventional drug carriers, nano-carriers for drugs have many advantages, such as smaller size, larger specific surface area, higher and more reactivity activity center, stronger adsorption capacity and other characteristics [17, 18]. (l) Drug-loaded nano-particle carriers targeted delivery of drugs to specific organs by modifying antibodies, aptamer, folic acid and other biological molecules. (2) Nano-particle carriers have relatively unique mode of controlling and releasing drugs. At the beginning, the mode of controlling and releasing drugs of nano-particle carriers is generally an outbreak release, and then it shows a constant release for a long period of time. Therefore, nano-carriers for drugs can significantly extend the effectivity of drugs at limited concentration, deliver at fewer intervals, and lower doses and reduce side effects and the suffering of patients. (3) Since the drug-loaded nano-particle carriers have the property of adhesion and small particle size, it is possible to extend drug stay in local tissues or sites, increase contact area of drugs and improve the absorption and bioavailability of oral drugs. (4) Drug-loaded nano-particle carriers can prevent rapid degradation of drugs by the digestive enzymes in the body in the process of conveying improved stability and utilization of drugs. (5) Drug-loaded nano-particle carriers can alter the mechanisms of membrane transport and enhance the permeability of drugs in bio-film, and then improve absorption effects of the drugs in cells. (6) The nanoparticles as carriers can modify the original drug to enhance water-solubility or get targeted and sustained release function, thereby enhance the efficacy of anticancer and reduce side effects of drugs.

### Applications of Quantum Dot Nano-Carriers for Drugs

Ideal QD nano-carrier materials for drugs should have the following properties [19]: (1) no reaction with drugs, (2) high drug loading capacity and encapsulation efficiency, (3) appropriate preparation and purification method (4) good biocompatibility and low toxicity, (5) certain mechanical strength and stability and appropriate particle size and shape, (6) longer residence time in vivo. At present, the commonly used anticancer nano-carriers for drugs are mainly liposome, chitosan, silica nanoparticles, and polymer nanoparticles.

QDs have unique optical properties, due to their quantum effect and size effect. When the particle size is of nanometer scale, it will cause quantum confinement effect, size effect, dielectric confinement effect, macroscopic quantum tunneling effect, and surface effect. Consequently, QDs exhibit many optical properties, and have a very broad application prospect in biological fluorescent probes and functional materials. Therefore, QDs will have a meaningful effect on the continued development of life sciences.

To our knowledge, an important aspect of developing modified QDs in biomedical applications is their selective targeting. Generally, in recent years, tumor cell targeting in both therapeutic and diagnostic applications have only focused on a small number of candidate ligands whose receptors are over-expressed in tumor cells, such as folic acid and delivery of siRNA. One such receptor is folic acid, which has widely been used as a targeting ligand to deliver therapeutic agents to cancer cells due to its high binding affinity with folate receptor (FR). And, studying siRNA delivery in cells and small animals using QDs should be an excellent choice because of QDs’ intrinsic fluorescence and their unique optical properties (e.g., tunable emission, photostability, and brightness).

### Folic Acid-Quantum Dot Complexes

**Fig. 1:**
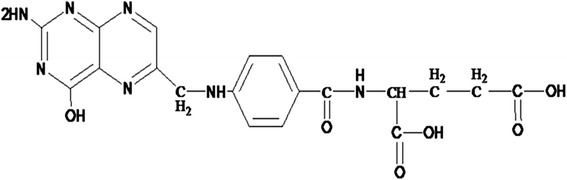
The scheme of folic acid

Folate (folic acid, FA), which is a donor number of carbon unit in vivo, is a necessary material to biosynthesize nucleic acids, amino and pantothenic acid, and its structure is shown in Fig. 1. FA is a needed vitamin for everyone, and it not only can participate in a variety of metabolic pathways of one-carbon transfer reactions, but also is the targeting ligand of FR. With the development of molecular biology and molecular medicine and in-depth study of tumor molecular level, researchers have found a series of receptors associated with tumor growth on the surface of tumor cells or tumor-associated blood vessels, and found the receptors high affinity to combine with its ligands. Therefore, ligands as targeting molecules of drug carriers can enhance therapeutic efficacy via a receptor-mediating mechanism, and then to achieve targeted therapy. FR is highly expressed in most tumor cells (such as ovarian cancer, cervical cancer, endometrial cancer, breast cancer, colon cancer, lung cancer, nasopharyngeal carcinoma choroid, and ependymal cell tumor cells), so as a low molecular weight targeting molecule, FA quickly becomes a hot topic of research [20]. Studies have shown that FA complexes modified by carboxyl deuterogenic remained a strong binding capacity with FR [21]. Typically, protein toxins, small molecule chemotherapeutic agents, radio-therapeutic agents, polymer-wrapped drugs, gene carriers, pro-drug inhibitors, and immunotherapeutic agents can form multimeric complexes bound with FA by covalent coupling. These FA complexes have better targeting ability and more therapeutic effect than the original drug, so the FA complexes have higher potential for drug delivery system [22, 23].

**Fig. 2:**
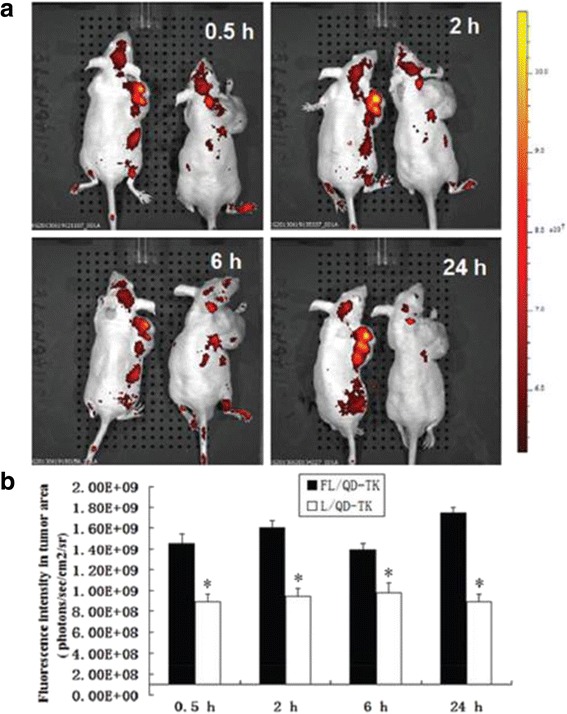
Real-time imaging of FL/QD-TK in Bel-7402 tumor xenograft model [24]

**Fig. 3:**
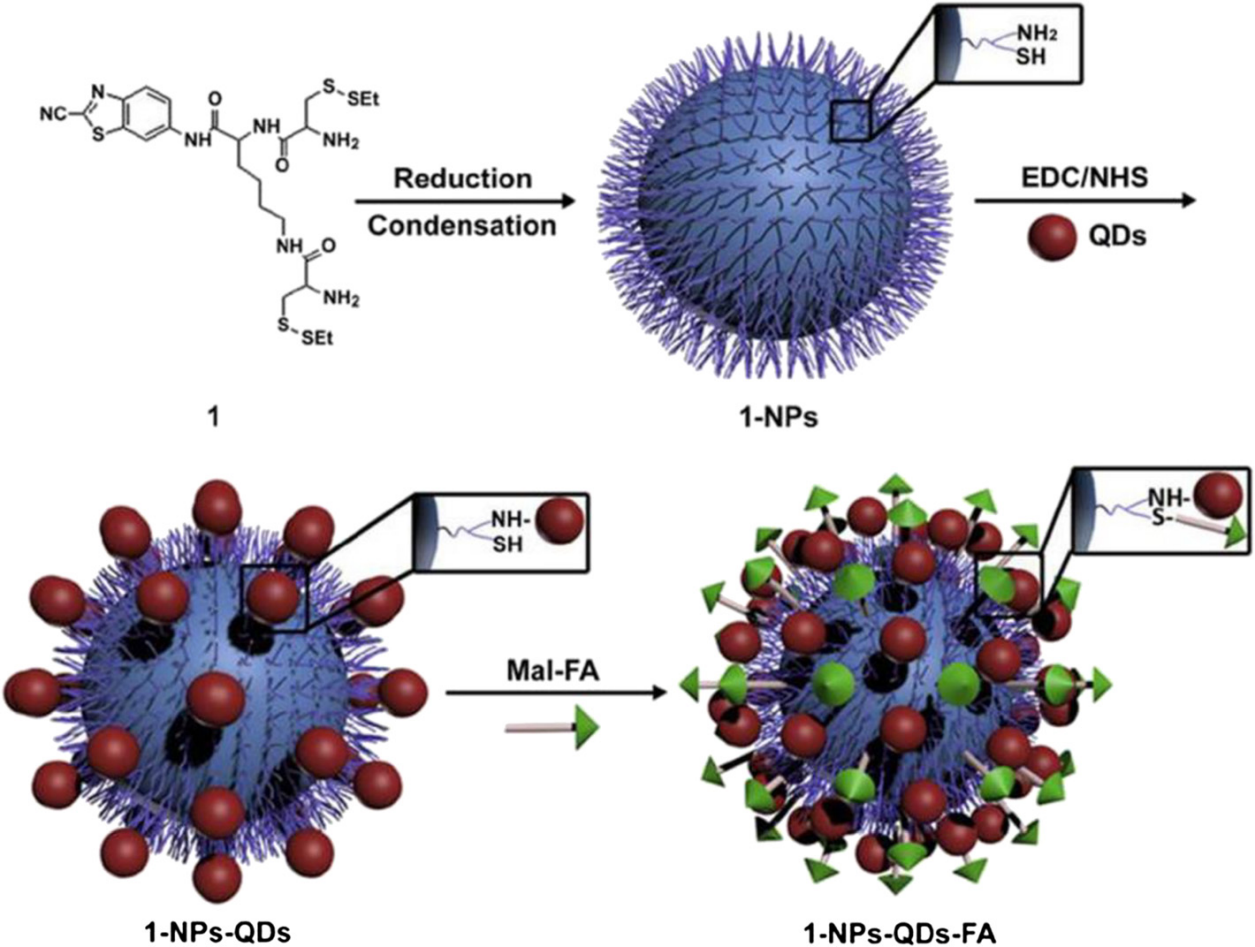
Schematic illustration of the preparative procedures of 1-NPs-QDs-FA [25]

FA, as a guiding molecule of anti-tumor drugs, has many advantages, such as low cost, high chemical stability, good receptor affinity, small size conducive to enter tumor cells through blood vessels, and completely infiltrate tumor tissues, etc., besides, FA complexes can well target to tumor cells. Now FA complexes have shown obvious advantages in targeting diagnosis and treatment on cancer. HSV-TK/GCV suicide gene systems are combined with near-infrared QDs, and the former is quite effective in liver cancer treatment, the latter facilitates tumor imaging [24]. A folate-modified theranostic (FL/QD-TK) was composed of an HSV-TK suicide gene covalently coupled with near-infrared fluorescent CdSeTe/ZnS core/shell QDs. FL/QD-TK exhibited highly specific tumor imaging and strong inhibition of the FR-over-expressed Bel-7402 mouse xenografts without systematic toxicity (Fig. 2) [24]. The facile surface-functionalization of one type of biocompatible, oligomeric nanoparticles 1-NPs binds with NIR-emitting CdTe/CdS QDs and folate could obtain tumor-targeted imaging in vivo (Fig. 3) [25]. As-prepared nanoparticles showed excellent targeting property to FR-expressing tumor cells in vitro and were successfully applied to selectively imaging FR over-expressing tumors in vivo.

**Fig. 4:**
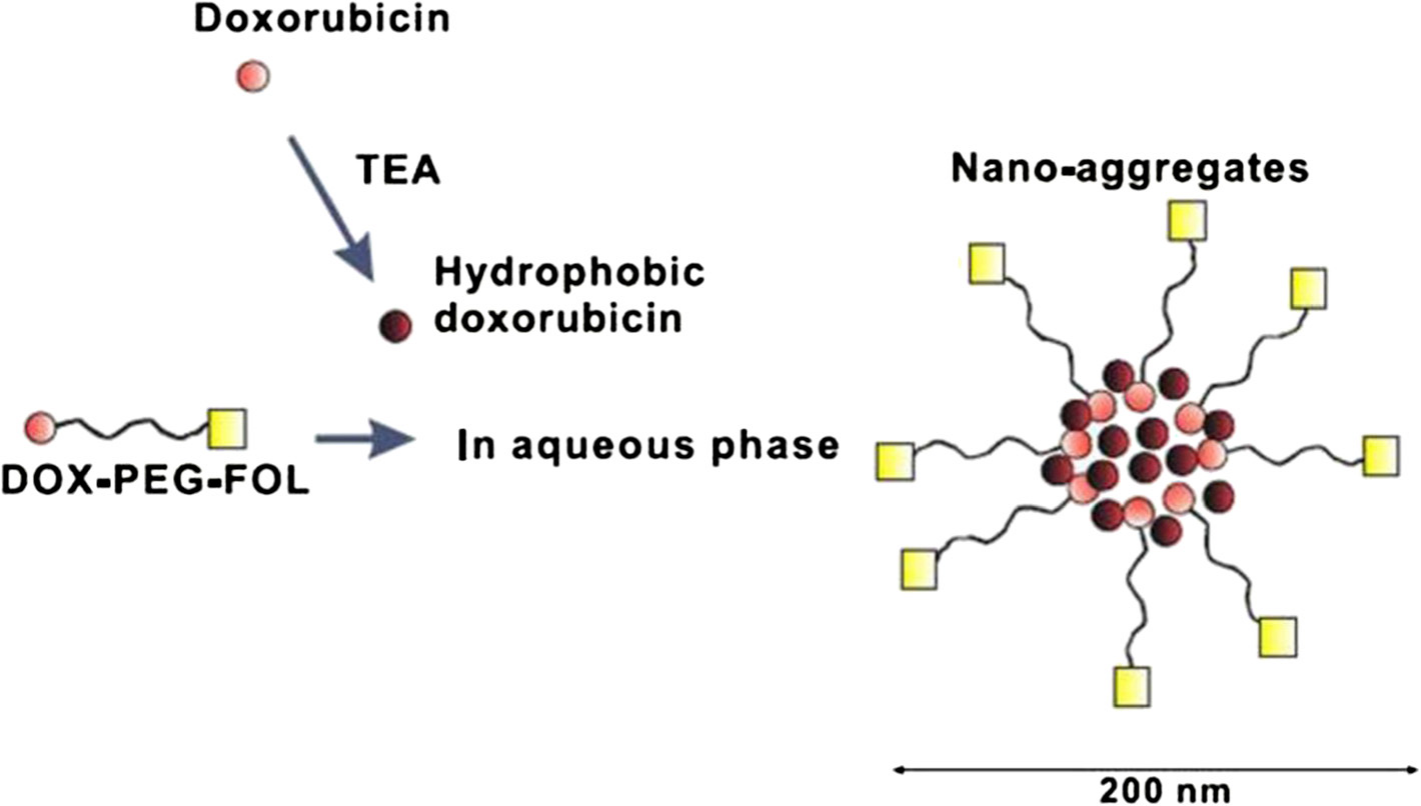
A scheme of folate-targeted DOX nano-aggregates with DOX-PEG-FOL conjugate, *FOL* folate [26]

Currently, as the tumor diagnosis materials, FA complexes have made some breakthroughs. For example some FA complexes coupled with radioactive elements (Fig. 4) have been used for clinical diagnosis of ovarian cancer [26]. But, the anti-tumor research about FA complexes in vivo was just the beginning. And, because FA complex structure and composition are complex, their metabolism and excretion in vivo, physiological function, stability, and toxicity are uncertain. In addition, some researchers consider that the release and treatment effects of drugs in FA complexes remain unsatisfactory in tumor tissues, so a lot of researches should be done before the FA complexes are used in clinical treatment of cancers. Along with widespread applications of QDs in biomedicine, more different fluorescent QDs made of FA complexes would be prepared for tumor diagnosis.

### Quantum Dots in RNA Interference (RNAi) Applications

Since RNAi was reported in *Caenorhabditis elegans*, RNAi phenomena were found in many organisms, such as zebrafish [27], fungi [28], drosophila [29], and mammalian mouse embryos [30]. However, the RNAi phenomenon was not found in archaea and prokaryotes; thus, it is possible that the RNAi is a means for advanced bio-specific to regulate gene expression and resist viruses or inhibit transposon-induced mutations [31, 32].

Small interfering RNA (siRNA) is quickly becoming a new tool for gene functional research and the new means of treatment [33]. After binding siRNA duplexes, RNA-induced silencing complex (RISC) was cleaved single-stranded siRNA. The targeted homology mRNAs binded with siRNA single strand are sheared by RISC to achieve the purpose of gene silencing. Now siRNA can enter cells like ribozyme by chemical synthesis or express short hairpin-like RNA (shRNA) by a carrier, and the latter can be transformed into siRNA in the cell to silence the related gene. Some studies have shown that there are other siRNA-silencing mechanisms, for example, siRNAs can lead to transcriptional gene silencing by RNAi-modified cellular chromatin in biology [34, 35]. RNAi as a new method of gene therapy has aroused the interest of many researchers [36], mainly because of low toxicity and specificity of RNAi, which is an endogenous regulation of gene expression substance in cells, and the other reason is that RNAi has higher gene silencing efficiency than that of ribozyme.

**Fig. 5:**
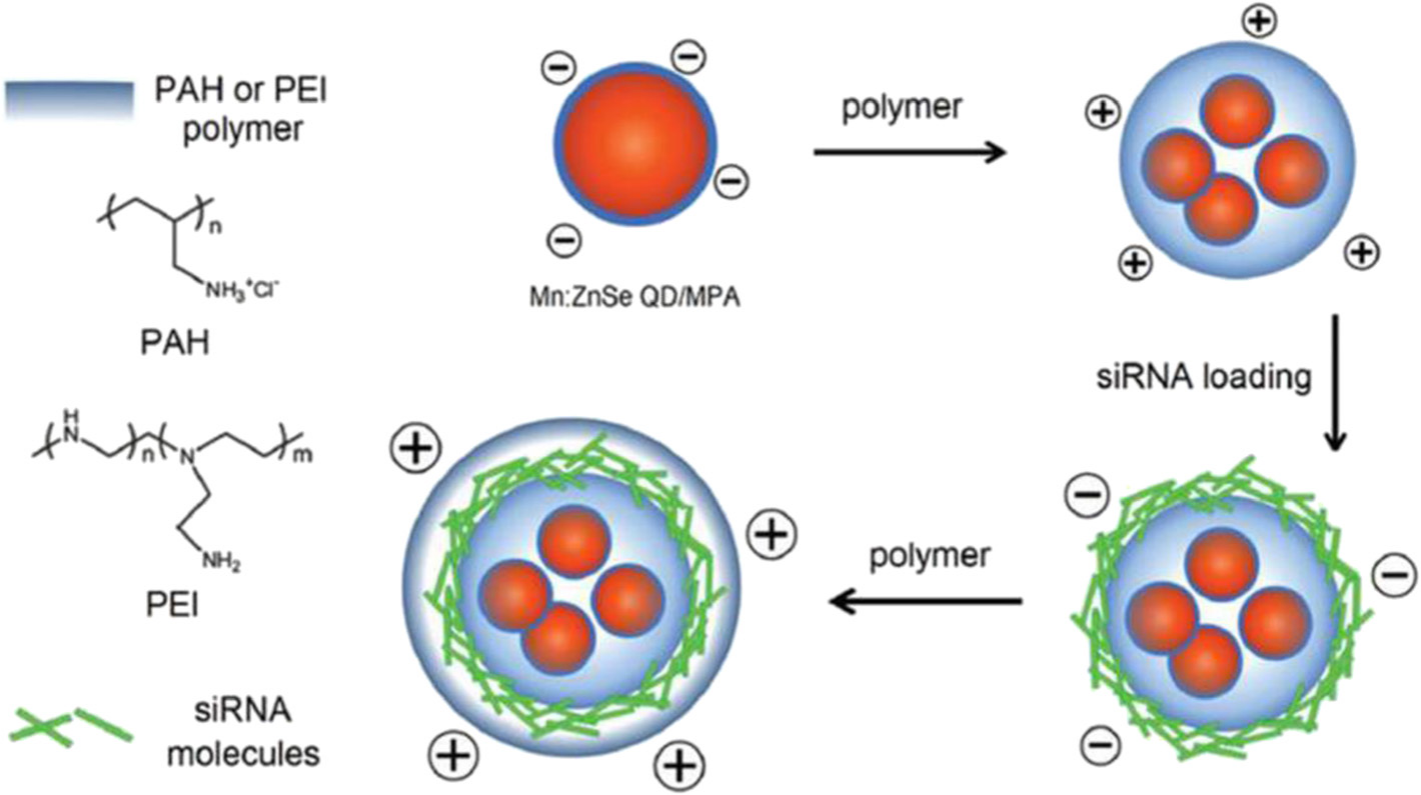
Schematic illustration of preparation steps of the Mn:ZnSe QD-based siRNA carriers [39]

The QD delivery systems are widely used to carry and image on siRNA in vivo and in vitro due to their inherent excellent optical properties. QDs are the ideal tool for discovering and validating in cells and small animals, but their potential uses in humans as drug delivery vehicles are unclear at present because bio-conjugated QDs cannot be efficiently cleared from the body either as intact particles or as ions [37, 38]. The large surface area of the amine-terminated nano-complex presents plenty of opportunities for further bio-functionalization while maintaining a high siRNA loading efficiency [37]. For example, the Mn:ZnSe d-dot can be used as a biocompatible nano-carrier for gene delivery in vitro (Fig. 5) [39]. Using a d-dot/polymer nano-complex as a transfection agent, siRNAs targeting the mutant oncogenic K-Ras gene were delivered into pancreatic cancer cells for sequence-specific gene therapy. The prepared nano-complex formulation achieved high gene transfection efficiency. Therapeutic effect was confirmed by the suppressed expression of the mutant K-Ras gene at the mRNA level. And, the d-dot/PAH nano-complex formulation is highly biocompatible even at a concentration as high as 160 μg mL^−1^, so the d-dots can act as a promising candidate for biomedical applications. And, the nano-complex can be functionalized with FA for receptor mediated cancer cell targeting and gene delivery.

**Fig. 6:**
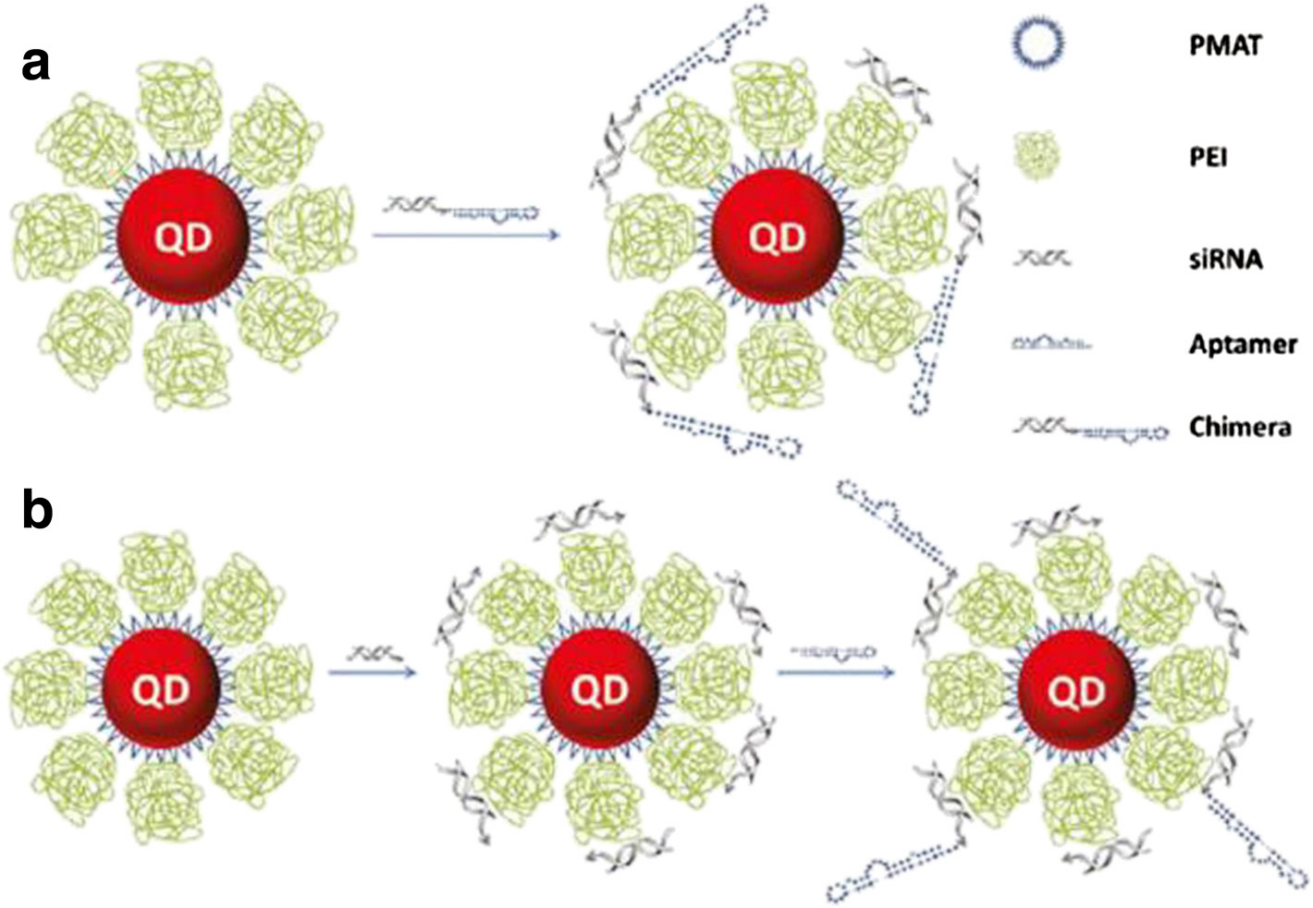
Schematic representation of cationic nanoparticles for targeted delivery of siRNA-aptamer chimeras [40]

SiRNA-aptamer chimeras are emerging as a highly promising approach for cell-type-specific delivery of siRNA due to the outstanding targeting capability of aptamers and the compatibility of chimeras with native ribonuclease (Dicer) processing [40]. For efficient RNAi, some challenges must be addressed, for example, how to get siRNA from the endosome after entering cells and how to retain aptamer targeting specificity when chimeras are combined with delivery carriers. Since both siRNA and aptamer are RNA molecules and often share similar molecular weight, so it is hard to design cationic delivery vehicles that selectively bind to the siRNA, leaving the targeting exposed aptamer. A rationally designed nano-particle carrier that simultaneously displays large surface area for high siRNA payload, exposed aptamer for specific targeting, proton sponge effect for endosome escape, and fluorescence for imaging and quantification were reported (Fig. 6) [40]. This method improved gene silencing efficiency over the conventional approaches based on simple mixing of siRNA-aptamer chimeras with cationic nanoparticles. This remarkable difference in RNAi efficiency using nano-particle-chimera complexes is directly related to cell uptake discrepancy resulting from aptamer conformation on nano-particle surface (intact vs. random).

In recent years, along with the emergence of stable expression carriers, the improvement of transfection efficiency, siRNA can stably express after transfecting and screening and can maintain interference effect for a long time, so that the applications of siRNA are more widely in functional genomics and gene therapy. However, there are some challenges about siRNAs used in clinical treatment, such as how to use the best way to import siRNAs intracellular, how to get more efficiency, and how to avoid missing target phenomenon and nonspecific reactions. Therefore, further study in RNAi mechanism will not only enrich our understanding of the regulation of gene expressions, but also facilitate practical applications of gene therapy.

### The Use of Duantum Dots in Studying Drug Release

Traditional methods of studying nano-drug release in vivo mainly determine the total content of drugs by HPLC-MS and other means, but these methods are often problematic at actual work. For example, it is difficult to quantify free drugs and encapsulated drugs separately, and there are technical difficulties in quantifying some trace drugs under the background of complex organisms. Recently, the QD-FRET (Förster resonance energy transfer) method provides a new way to solve these problems. The principle of QD-FRET method is when the fluorescence emission spectrum of QDs and the excitation spectrum of fluorescent receptors overlap and the spatial distance between the donor and acceptor is close enough, the QDs at states of excitation can be used as a fluorescence donor to transfer fluorescence energy to the acceptor, which results in fluorescence quenching of the QDs donor and fluorescence enhancement of the acceptor. This energy transfer usually occurs when the distance of intermolecules is 1–10 nm, and the efficiency of energy transfer closely relates to the distance between each other, namely, the smaller the distance, the higher the FRET efficiency. This technology has been successfully applied in interaction studies of biological macromolecules, immunoassay, nano-sensor design, and other fields [41, 42]. Recently, the researchers have made some progress at nano-drugs release [17].

**Fig. 7:**
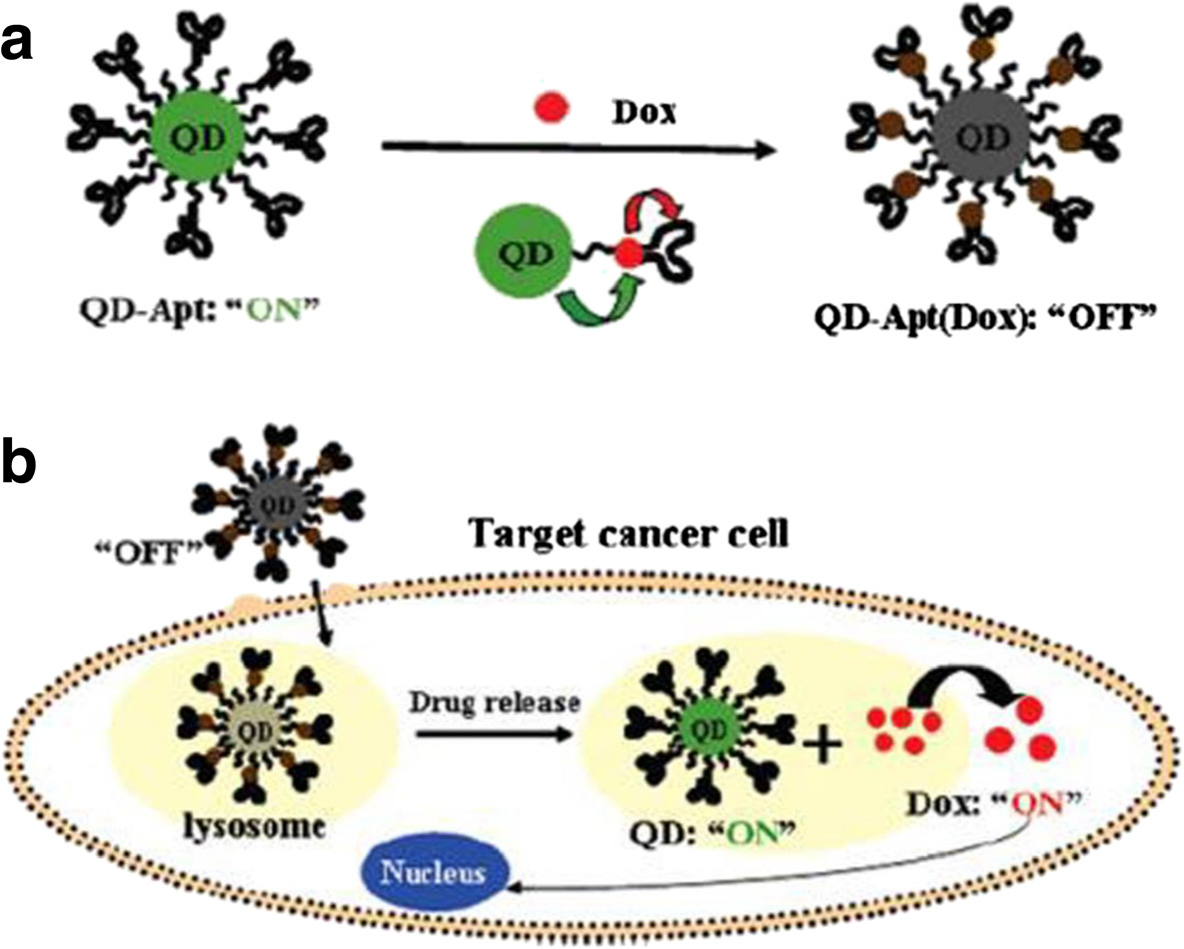
Schematic illustration of QD-aptamer (Dox) Bi-FRET system [43]

In addition, the two-FRET system of QDs could release Dox in cells (Fig. 7) [43]. The identified prostate-specific membrane antigen A10 RNA aptamer were coated on the QD surface, and then they were mixed with Dox solution to make Dox inserted into the inner aptamer duplex to form QD-aptamer (Dox) complexes. The donor-acceptor consisting of Dox and QDs by cutting role and the donor-quenching body double FRET system consisting of Dox and aptamer resulted in reversible fluorescence self-quenching of complexes. With the extension of incubation time, and after the complexes are recognized and taken by prostate tumor cells, Dox is released from the complexes, and the fluorescence signals of QDs and Dox are significantly increased in cells. Both tumor cell localization and intracellular Dox release testing can be achieved by measuring the fluorescence signal changes.

A wide variety of both water-soluble and insoluble drugs can be incorporated into nanoparticles with a high efficiency and modifiable drug loadings. Using QDs as drug models and imaging labels to evaluate nano-particle formulations incorporating both hydrophilic and hydrophobic drugs and imaging agents is developed. The use of QDs enables efficient detection and precise quantization of cellular uptake of particles. The correlation of QDs- and doxorubicin-incorporated nanoparticles is useful to develop an evaluation platform for nano-particle formulations through imaging and quantization [44]. QDs can be used as a model for such non-fluorescent drugs, and QD-PNPs can be used as a model to monitor the position of used polymer and evaluate the efficacy of the proposed delivery system. Moreover, QDs can be used as visible ring surrounding the micro-particles to study micro-particle theranostic delivery formulations by deliberately incorporating, for they are easy to be identified and traced in diagnostic and chemotherapeutic applications.

**Fig. 8:**
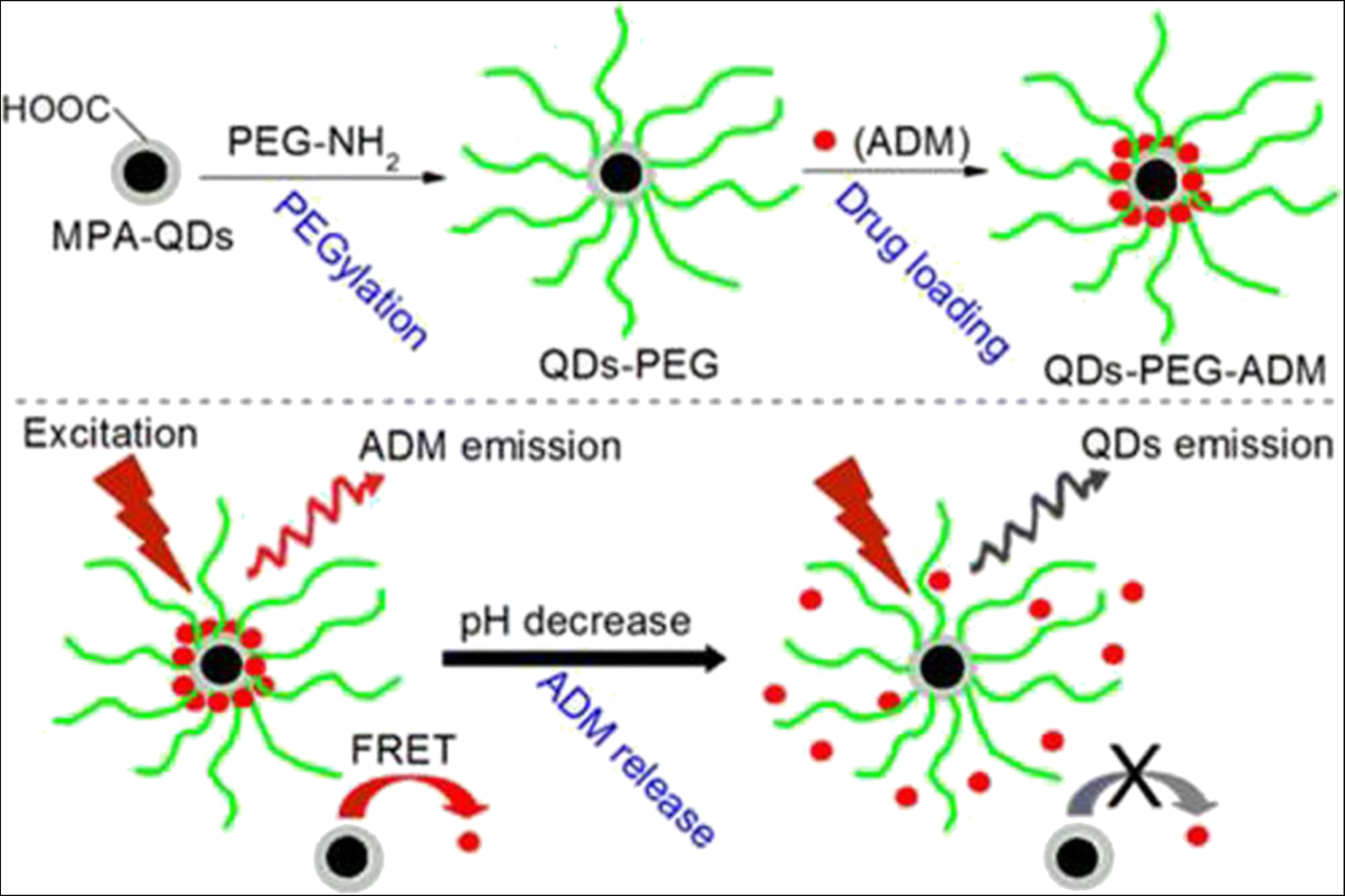
Schematic of the preparation of QD-PEG-ADM and the mechanism of this system for drug release [45]

An effective and facile FRET-based QD-PEG-Dox drug delivery system (DDS) has been used for real-time monitoring of the release of loaded drugs (ADM) in situ (Fig. 8) [45]. An outstanding correlation between the FRET signal and drug release in different pH environments has been applied to real-time detection of (Dox). The methods of ratiometric and time-resolved fluorometry are practical for real-time monitoring of drug release in tumor cells. It would promote the development of versatile DDS, and offering a sensitive and efficient method to monitor in real-time the release in situ of drugs, especially in physiological microenvironments.

## Conclusions

Although the research of QD nano-carriers for drugs has achieved some developments, the application of QD nano-carriers for drugs is still at the beginning. Using the QD nano-carriers for drugs in biology is a burgeoning new field but has high value. Along with the continuous development of the technique of QD nano-carriers for drugs, new development opportunities for drug screening, disease screening, and gene sequencing, plurality of biomedical research will come.
